# Transcriptome sequencing, molecular markers, and transcription factor discovery of *Platanus acerifolia* in the presence of *Corythucha ciliata*

**DOI:** 10.1038/s41597-019-0111-9

**Published:** 2019-07-22

**Authors:** Fengqi Li, Chunyan Wu, Mengzhu Gao, Mengmeng Jiao, Cheng Qu, Asier Gonzalez-Uriarte, Chen Luo

**Affiliations:** 1 Institute of Plant and Environment Protection, Beijing Academy of Agriculture and Forestry Sciences, Beijing, 100097 China; 2 College of Life Sciences, Capital Normal University, Beijing, 100037 China; 3 Rothamsted Research, Computational and Analytical Sciences Department, Harpenden, AL5 2JQ UK

**Keywords:** Forestry, Invasive species, Biotic, RNA sequencing

## Abstract

The London Planetree (*Platanus acerifolia*) are present throughout the world. The tree is considered a greening plant and is commonly planted in streets, parks, and courtyards. The Sycamore lace bug (*Corythucha ciliata*) is a serious pest of this tree. To determine the molecular mechanism behind the interaction between the London Planetree and the Sycamore lace bug, we generated a comprehensive RNA-seq dataset (630,835,762 clean reads) for *P*. *acerifolia* by sequencing both infected and non-infected leaves of *C*. *ciliata* using the Illumina Hiseq 4000 system. We assembled the transcriptomes using the Trinity De Novo assembly followed by annotation. In total, 121,136 unigenes were obtained, and 80,559 unigenes were successfully annotated. From the 121,136 unigenes, we identified 3,010,256 SNPs, 39,097 microsatellites locus, and 1,916 transcription factors. The transcriptomic dataset we present are the first reports of transcriptome information in *Platanus* species and will be incredibly useful in future studies with *P*. *acerifolia* and other *Platanus* species, especially in the areas of genomics, molecular biology, physiology, and population genetics.

## Abstract (summary)

**Table:** 

Design Type(s)	transcription profiling design • sequence assembly objective • sequence annotation objective
Measurement Type(s)	transcription profiling assay
Technology Type(s)	RNA sequencing
Factor Type(s)	experimental condition • temporal_interval
Sample Characteristic(s)	Platanus × hispanica • leaf

Machine-accessible metadata file describing the reported data (ISA-Tab format)

## Background & Summary

Transcriptional sequencing technology is used in biological research for the gene expression profile investigation, the biological molecular evolution, and molecular marker acquisition^1–4^. The technology is particularly convenient for non-model organisms, for which there is no genome data available^5,6^. Abundant transcriptome data of some garden trees are reported as the demand for continuous development of urban landscaping^7–9^.

The London Planetree (*Platanus acerifolia*) is a hybrid cross between the American sycamore (*P*. *occidentalis*) and the Oriental Planetree (*P*. *orientalis*)^10^. *P*. *acerifolia* is a woody arbor plant with a large crown that grows rapidly, provides dense shade, and is tolerant to urban pollution^11^. This species is commonly grown around the world and is known as “the king of street trees^12^”. Despite its widespread use, there is a lack of research regarding the molecular biology of the tree, and there are no publicly available genome or transcriptome resources for the species or the genus. For this reason, research on genetic diversity and work on genetic engineering using molecular biotechnology is limited.

A particularly harmful pest to *P*. *acerifolia* is the sycamore lace bug (*Corythucha ciliata*), which is native to North America but was introduced to Europe in the 1960s^13^. The bug was first found in Hunan province in China in 2002 and has since spread to Hubei, Shanghai, Shandong, Henan, and Beijing, where heavy infestations have been reported^14,15^. The sycamore lace bug specifically damages *Platanus* trees, causing chlorotic or bronzed foliage and premature senescence of leaves^16^. Currently, transcriptome resources are not available for the genus *Platanus*, even though such data would deepen our understanding of the interaction mechanism between *P*. *acerifolia* and *C*. *ciliata* and promote related research between in the two other *Platanus species*.

The objectives of our study were to determine the leaf transcriptome dataset of this tree. The leaf transcriptome of *P*. *acerifolia* was sequenced on the Illumina HiSeq 4000 platform, and 637,324,886 raw reads were generated. After filtering reads of low quality, the 630,835,762 clean reads were assembled de novo and led to 121,136 unigenes. A total of 76,203, 52,758, 48,527, 8,849, 57,997, and 34,193 unigenes were annotated with a significant Blastx against non-redundant (Nr), SwissProt, Protein family (Pfam), Clusters of Orthologous Groups (COG), gene ontology (GO), Kyoto Encyclopedia of Genes and Genomes (KEGG) databases, respectively. After transcriptome sequences, molecular marker and transcription factor were mined. A total of 3,010,256 single nucleotide polymorphisms (SNPs) were identified in all samples, and 39,097 microsatellites (simple sequence repeats, SSR) were identified cross the 121,136 unigenes. In addition, 1,916 transcription factors were identified. This data descriptor provides an opportunity to identify the functional genes and molecular marker for *P*. *acerifolia*. This comprehensive *P*. *acerifolia* transcriptomic information can be utilized to promote the insect defense mechanisms in *P*. *acerifolia*.

## Methods

### Material treatment

Leaf samples of *P*. *acerifolia* were collected from mature trees that were in the courtyard of Beijing Academy of Agriculture and Forestry Sciences (Beijing, China) during July 2017 (Table 1). Only healthy leaves were selected. The leaves, including the petiole, were detached from the tree and placed in a glass tube with 10 mL sterile water. The glass tubes were sealed with absorbent cotton and placed in a 2 L glass beaker. Each leaf was inserted into 100 *C*. *ciliata*, which were raised according to previous research^16^. The experiments were performed in a growth chamber (25 ± 2 °C, 50–70% RH, 16:8 L:D). The insects on the leaves were treated for 24 h, 48 h and removed with a soft brush. Control leaves (control) were grown as the others but without *C*. *ciliate* infestation. After treatment, each plant leaf sample was collected for RNA extraction. Each treatment was performed in three biological replicates.

**Table 1:** Characteristics of the *Platanus acerifolia* transcriptome sequencing project.

Item	Description
Investigation type	Eukaryote transcriptome
Sampling date	5 July 2017
Geographic location	9°56′32.60″N E116°16′53.73″E
Tissue type	Leaves
Sequencing technology	Illumina Hiseq 4000
Assembly	Trinity
Finishing strategy	Contigs
Data accessibility	SRP156640

### RNA isolation, cDNA library, and illumina sequencing

Total RNA was extracted using the TRIzol reagent (Invitrogen, CA, USA). The integrity and the purity of total RNA were verified using an Agilent Bioanalyzer 2100 and RNA 6000 Nano LabChip Kit (Agilent Technologies, CA, USA) with a minimum RNA integration number of 7. Approximately 10 μg of the total RNA representing a specific adipose type was subjected to isolate Poly (A) mRNA with poly-T oligo-attached magnetic beads (Invitrogen, CA, USA). After purification, the poly(A)− or the poly(A)+ RNA fractions were fragmented into small pieces using divalent cations under elevated temperatures. The cleaved RNA fragments were reverse-transcribed to create the final cDNA library in accordance with the protocol for the mRNA-Seq sample preparation kit (Illumina, San Diego, USA). The average insert size for the paired-end libraries was 300 bp (±50 bp). The paired-end sequencing was performed on an Illumina Hiseq 4000 following the vendor’s recommended protocol.

### De Novo assembly, unigene annotation, and functional classification

Fastp^17^ was used to remove the readings that contained adaptor contamination, low quality bases, and undetermined bases. The sequence quality was verified via FastQC (http://www.bioinformatics.babraham.ac.uk/projects/fastqc/), including the Q20, the Q30, and the GC-content of the clean data. The downstream analyses were based on high-quality clean data. De Novo assembly of the transcriptome was performed with Trinity 2.4.0^18^. Next, TransRate^19^ and BUSCO^20^ were used to assess De Novo transcriptome assembly quality. The assembled unigenes were aligned against the Nr protein (http://www.ncbi.nlm.nih.gov/), Pfam, COG, and the SwissProt (http://www.expasy.ch/sprot/) databases using BLASTx^21^ with an E-value threshold of <0.00001. The gene ontology (GO) annotations were obtained using Blast2GO^22^ (http://www.blast2go.com/b2ghome). Metabolic pathway analysis was performed using the Kyoto Encyclopedia of Genes and Genomes (KEGG, http://www.genome.jp/kegg/)^23^.

### SNPs, SSRs, and transcription factor identification

SAMtools package^24^ was used to detect potential SNPs. SNPs were filtered based on the following criteria: (1) the number of reads to cover a candidate SNP above 8; (2) remove low quality where base calls with low Phred quality below 25; (3) frequency of mutated bases among all reads covering the position above 30%. For all unigenes, SSRs were identified using MISA^25^ (http://pgrc.ipk-gatersleben.de/misa/misa.html) according to default parameters, and the primer for each SSR was designed by Primer3 (http://primer3.sourceforge.net/releases.php)^26^. The transcription factor families were identified using the Plant Transcription Factor Database PlantTFDB 4.0 (http://planttfdb.cbi.pku.edu.cn/prediction.php)^27^.

## Data Records

The annotation, molecular markers, and transcription factor output files were provided in Figshare^28^. Raw FASTQ files for the RNA-Seq were deposited to the NCBI SRA database under SRA accession number SRP156640^29^. The final assembled unigenes sequences were deposited at NCBI GenBank (GGXZ00000000.2)^30^.

## Technical Validation

High throughput sequencing generated 46,890,842–57,342,752 pairs of raw reads per sample^29^, and the Q20 scores (the average quality value) were greater than 97%. The GC content of clean reads was similar, ranging from 46.14% to 47.36% (Online-only Table 1). The total length of the combined reads for the 12 samples that represented the different stages of damage was 202,095,905 bp and 121,136 unigenes^28^; the average length was 1015.15 bp with an N50 of 1579 bp and an E90N50 of 1762 bp (Table 2).

**Table 2:** Assembly information of the *Platanus acerifolia* transcriptome dataset.

Type	Resource
Total transcripts number	199,080
Total unigenes number	121,136
Total sequence base	202,095,905
Largest	18,931
Smallest	201
Average length	1015.15
N50	1579
E90N50	1762
GC percent	41.50
Mean mapped reads	1442.7636918
TransRate score	0.25922
BUSCO score	72.1% (3.3%)

All 121,136 unigenes found in *P. acerifolia* leaves were functionally annotated using six public databases (Table 3). Of unigenes, 62.91% (76,203) were annotated to the NR database, 43.55% (52,758) were annotated to proteins in the Swiss-Prot database, 40.06% (48,527) were annotated to proteins in the Pfam database, 7.31% (8,849) were annotated to the COG database, 47.88% (57,997) were annotated to the GO database, and 28.23% (34,193) were annotated to the nucleotide sequences in the KEGG database. In total, 66.5% of unigenes (80,559) were annotated to a database.

**Table 3:** Annotation information of the *Platanus acerifolia* transcriptome dataset.

	Unigene number (%)
NR	76,203 (62.91)
Swiss-Prot	52,758 (43.55)
Pfam	48,527 (40.06)
COG	8,849 (7.31)
GO	57,997 (47.88)
KEGG	34,193 (28.23)
Total_annotation	80,559 (66.5)
Total	121,136 (100)

**Fig. 1:**
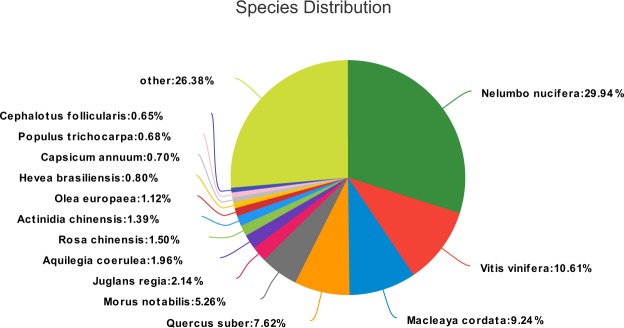
Species distribution of the NR annotation.

The similarity analysis of the NR database demonstrated that there were 39,436 unigenes with significant homology (E-values < 1e^−30^) to other sequences in the Nr database and 36,767 unigenes with E-values between 1e^−5^ and 1e^−30^. The NR annotation species distribution analysis showed that 22,670 unigenes had higher homology with *nelumbo_nucifera*, which accounted for 29.94% of the total (Fig. 1)^28^. In addition, Swiss-Prot and Pfam annotation results were deposited in Swiss-prot_annotation.xls and Pfam_annotation.xls, respectively^28^.

**Fig. 2:**
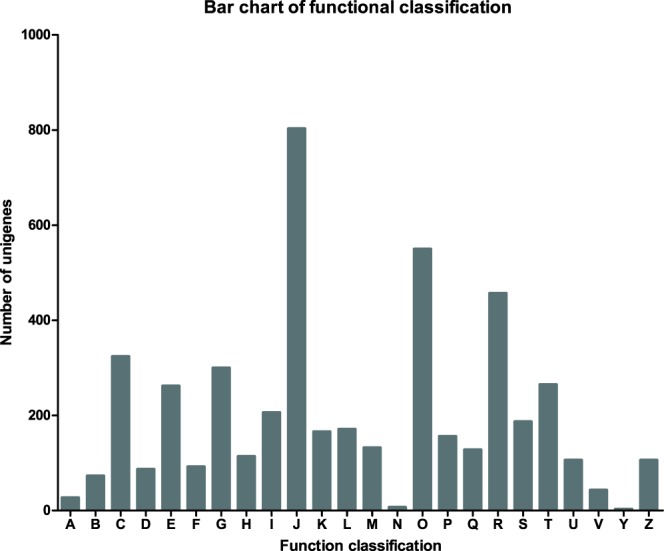
The COG functional categories.

After COG based annotation, a total of 8,849 unigenes were assigned to 24 functional categories (Fig. 2)^28^. For COG annotation, the two largest COG categories were “Translation, ribosomal structure, and biogenesis” (803, 16.85%) and “Posttranslational modification, protein turnover, chaperones” (550, 11.54%). The following abundant groups were “General function prediction only” (457, 9.59%), “Energy production and conversion” (324, 6.80%), “Carbohydrate transport and metabolism” (300, 6.30%), “Signal transduction mechanisms” (265, 5.56%), and “Amino acid transport and metabolism” (262, 5.50%). The two groups involving “Cell motility” (7, 0.147%) and “Nuclear structure” (3, 0.063%) represented the smallest COG classifications. Lastly, 43 unigenes (0.902%) were classified into “Defense mechanisms”.

**Fig. 3:**
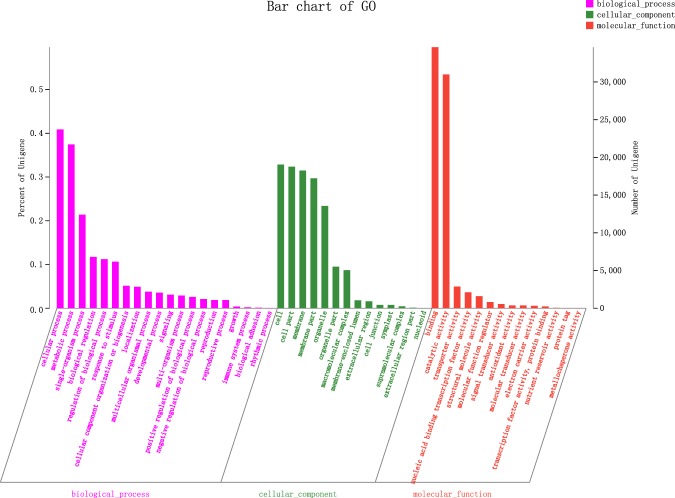
GO classification of the *Platanus acerifolia* unigenes.

A total of 57,997 unigenes were annotated in the GO database, 53.14% (29,079) for the biological process, 58.80% (49,763) for the molecular function, and 56.13% (32,553) for the cellular component. The categories “cellular process,” “metabolic process,” and “single-organism process” were most abundant among the biological process GO category. Within the cellular component category, the “cell” and “cell part” terms were most abundant. For the molecular function, the unigenes were chiefly related to “binding” and “catalytic activity” (Fig. 3)^28^.

**Fig. 4:**
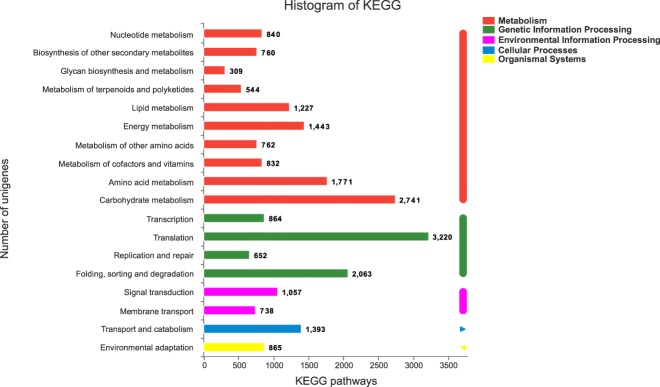
KEGG pathway distribution of the *Platanus acerifolia* unigenes.

We mapped the unigenes to the reference authoritative pathway in KEGG for further functional classification and annotation. In total, 34,193 unigenes were distributed among 130 KEGG pathways, and 11,229 (32.84%) were related to metabolic pathways. The largest number of unigenes involved were in the “Carbohydrate metabolism” (2741) category, followed by the “Amino acid metabolism” (1771) category, whereas “Glycan biosynthesis and metabolism” (309) was the smallest group (Fig. 4 and kegg_annotation.xls)^28^.

**Fig. 5:**
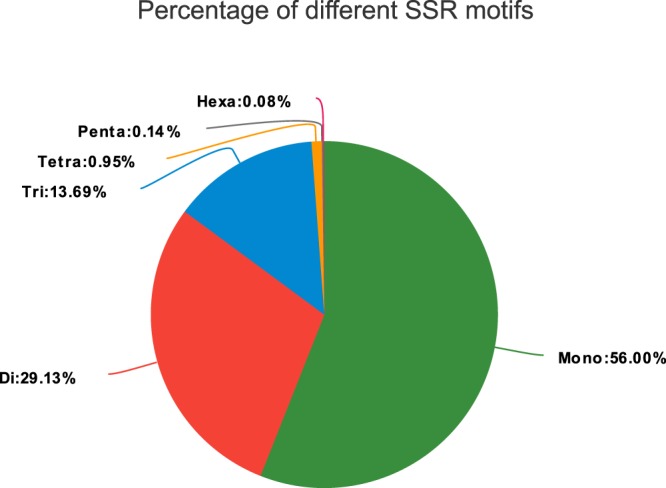
Percentage of different SSR motifs.

We screened the *P*. *acerifolia* unigene dataset to determine potential SNPs and SSRs for future populations and genetics analysis. Among unigenes sequences, we detected 28,144 unigenes containing SSRs and 6,053 unigenes containing more than one SSR. According to the repeat motif, the SSR loci can be divided into six categories: mono-nucleotide repeats (21,895), di-nucleotide repeats (11,388), tri-nucleotide repeats (5,353), tetra-nucleotide repeats (373), penta-nucleotide repeats (55), and hexa-nucleotide repeats (33) (Fig. 5, ssr_repeats.xls, ssr_analysis_details.xls)^28^.

A total of 3,010,256 SNPs were obtained from the twelve leaves samples. Among these SNPs, 1,503,269 and 1,506,987 SNPs were obtained from the CK and insect treated samples, respectively. And, 1,005,449 SNPs were homo-type, 2,004,807 were hete-type (snp_homo_hete_statistics.xls, snp_detail.xls)^28^. Among them, 1,349,858 were putative transitions, and 791,734 were putative transversions. The transition-type SNPs include four classes (A/G, C/T, G/A, and T/C) and the transversion-type SNPs include eight classes (A/C, A/T, C/A, C/G, G/C, G/T, T/A, and T/G). (snp_transition_tranversion_statistics.xls, snp_detail.xls)^28^.

**Fig. 6:**
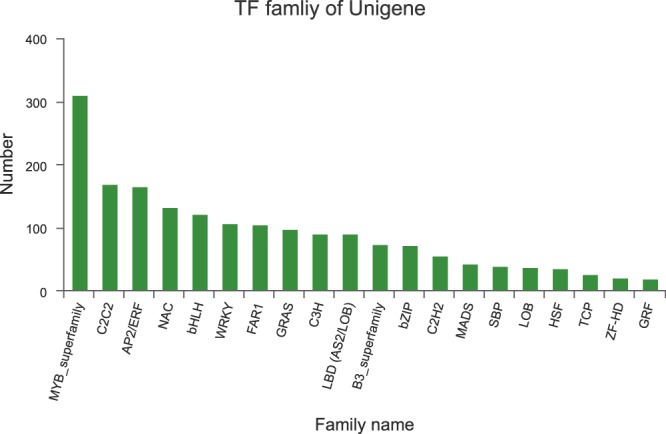
Transcription factor family of the *Platanus acerifolia* unigenes.

In order to promote functional gene research in *P. acerifolia*, we identified a series of transcription factors, which included 35 gene families. Among them, MYB_superfamily had as many as 311 unigenes, and C2C2 and AP2/ERF had 168 and 166 unigenes, respectively. NAC had 132 unigenes, bHLH had 122 unigenes, and both WRKY had 107 unigenes (Fig. 6, Transcription_Factor_annotation.xls)^28^.

The comprehensive datasets we present are the first reports of transcriptome information in *Platanus* species and will facilitate the identification of insect defense-related genes in the future. The annotated unigenes are a significant improvement on the sequence information available for *P*. *acerifolia* and other closely related species. The identified SNPs and SSR locus resources will be of help in population genetic structure, gene flow studies, and parentage analysis for *P*. *acerifolia*. The reported transcription factors in this dataset will be useful resources to further explore the physiological and biochemical mechanisms of growth development and stress response in *P*. *acerifolia* and other *Platanus* species.

### ISA-Tab metadata file

Download metadata file

## Data Availability

The following software were used for data analysis: 1. Fastp was used for preprocessing for FastQ files. https://github.com/OpenGene/fastp. 2. FastQC was used for quality control. http://www.bioinformatics.babraham.ac.uk/projects/fastqc/. 3. Trinity 2.4.0 was used to de novo transcriptome assembly. https://github.com/trinityrnaseq/trinityrnaseq. 4. Transrate v1.0.3 and BUSCO v3 were used for assessing assembly quality. http://hibberdlab.com/transrate/ and https://busco.ezlab.org/. 5. Blast2GO was used for GO annotation. http://www.blast2go.com/b2ghome. 6. KEGG database was used for metabolic pathway annotation. http://www.genome.jp/kegg/. 7. SAMtools was used for detecting SNPs. https://samtools.github.io/bcftools/howtos/variant-calling.html. 8. MISA was used to identify SSRs. http://pgrc.ipk-gatersleben.de/misa/misa.html. 9. PlantTFDB 4.0 database was used for predicting transcription factor. http://planttfdb.cbi.pku.edu.cn/prediction.php.

## Online-only Table

**Online-only Table 1:** Sequencing statistics for the *Corythucha ciliata* infection and the non-infected leaves of *Platanus acerifolia*.

Sample	Raw reads	Raw bases	Clean reads	Clean bases	Error rate (%)	Q20 (%)	Q30 (%)	GC content (%)
CK24h_1	57,342,752	8,658,755,552	56,754,744	8,464,801,247	0.0254	97.91	93.7	47.34
CK24h_2	54,566,682	8,239,568,982	54,031,744	8,064,184,328	0.025	98.1	94.13	46.27
CK24h_3	49,729,386	7,509,137,286	49,215,194	7,349,321,932	0.0258	97.76	93.31	46.13
CK48h_1	54,194,972	8,183,440,772	53,612,120	7,995,900,899	0.026	97.68	93.17	46.45
CK48h_2	46,890,842	7,080,517,142	46,481,784	6,938,620,927	0.0247	98.22	94.41	46.21
CK48h_3	55,931,148	8,445,603,348	55,345,842	8,252,377,339	0.0255	97.89	93.64	46.16
T24h_1	52,994,650	8,002,192,150	52,365,790	7,812,178,204	0.0259	97.71	93.22	46.34
T24h_2	49,989,010	7,548,340,510	49,508,518	7,398,086,642	0.0249	98.13	94.2	46.39
T24h_3	53,964,742	8,148,676,042	53,420,354	7,974,482,782	0.0252	97.99	93.87	46.4
T48h_1	55,717,534	8,413,347,634	55,181,552	8,233,891,794	0.0254	97.92	93.7	46.21
T48h_2	53,547,068	8,085,607,268	52,953,230	7,899,595,930	0.0256	97.84	93.52	46.3
T48h_3	52,456,100	7,920,871,100	51,964,890	7,753,024,901	0.0249	98.14	94.21	46.65
Total	637,324,886	96,236,057,786	630,835,762	94,136,466,925				
